# Potentials and pitfalls of including pharmacies as youth-friendly contraception providers in low- and middle-income countries

**DOI:** 10.1136/bmjsrh-2020-200641

**Published:** 2020-08-11

**Authors:** Lianne Gonsalves, Adriane Martin Hilber, Kaspar Wyss, Lale Say

**Affiliations:** 1 Department of Sexual and Reproductive Health and Research including UNDP/UNFPA/UNICEF/WHO/World Bank Special Programme of Research, Development and Research Training in Human Reproduction (HRP), World Health Organization, Geneva, Switzerland; 2 Swiss Centre for International Health, Swiss Tropical and Public Health Institute, Basel, Switzerland; 3 University of Basel, Basel, Switzerland

**Keywords:** family planning services, pharmaceutical Services, adolescent, contraception behavior

## Pharmacies enabling youth ‘self-care’ in sexual and reproductive health

Older adolescents and young adults between the ages of 15–24 years (collectively termed ‘youth’) face a variety of barriers when trying to access sexual and reproductive health (SRH) services – particularly contraceptive services – from health facilities.^1^ Policy restrictions and community norms which deem youth access ‘inappropriate’ can dissuade young people from going to a facility. Those who do go often encounter ‘unfriendly’ staff, and facilities with inconvenient opening hours or a lack of confidentiality and privacy.^1^

In recent years, evidence on the safety and efficacy of contraceptives, coupled with global commitments by countries and donors to address health systems barriers and expand access to contraception (eg, Family Planning 2020^2^), have resulted in key services and products being made available outside of health facilities.^3^ As a result, certain contraceptive products, including emergency contraception and daily contraceptive pills, are now available through retail pharmacies and drug shops (lower-tier establishments which do not employ a trained pharmacist, and are limited in the drugs they can dispense^4^).^5^ Additionally, the recent advance of self-administered injectable contraception reflects a trend towards broader ‘self-care’ (individuals addressing their own health needs, with or without the support of a healthcare provider) and user autonomy in accessing and using modern contraceptive methods.^6 7^

Pharmacies and drug shops, therefore, could be considered an important alternate contraception source for young people, especially in low- and middle-income countries (LMICs).^4^ Yet they are not uniformly lauded as a panacea to meet young people’s need for contraception. The breadth of contraception available in many pharmacies remains narrow. Also, many LMICs struggle with variable service quality and illegal pharmacy activity, including unregistered premises, untrained personnel and/or substandard commodities. So, to what extent can pharmacies and drug shops be a quality catalyst for empowering a young population around their SRH?

## ‘Youth-friendly enough’ – pharmacies as contraception providers to young people

Service provision to young people in general can be assessed by five tenets of quality, or adolescent/youth-“friendliness”: accessibility, acceptability, appropriateness, effectiveness and equity.^8^ From a programming perspective, *all* five qualities must be present to ensure quality services are available to all young people who need them. However, studies which have explored young people’s access to contraception services through pharmacies indicate that these standards may not be equally valued by young users.^9^

Appropriateness (ensuring the *right* health services are provided) and effectiveness (ensuring the right services are delivered in the correct manner) are essential for health programmers and governments. However, young people encountering individual, family and/or community resistance related to sexual activity value confidentiality and respectful treatment (both of which are elements of acceptability).^8^ It is unsurprising, therefore, that pharmacy services are also appreciated for their acceptability and accessibility: non-judgmental personnel (in certain settings), privacy (in certain settings), convenient locations and proximity, opening hours, speed of service and ease of access.^9^ As a result, young people seek support from pharmacies and drug shops, even where there are concerns about service appropriateness, effectiveness or cost. Put simply, young pharmacy customers prioritise a narrower set of ‘quality’ standards than health programmers or policymakers.

## Attention to equity – financing contraception services in pharmacies

Pharmacy personnel providing contraception services and broader support for ‘self-care’ brings contraception closer to users of all ages. Additionally, ‘self-care’ has generated enthusiasm for its potential to improve the cost-effectiveness of delivering SRH interventions. For pharmacy interventions, however, financing concerns remain: pharmacy services in many countries, particularly in LMICs, are often paid for out-of-pocket (a non-reimbursed, direct payment by the individual).

As identified by Remme and colleagues,^10^ evidence on how self-care interventions are financed is lacking, and with it insights into the equity (or lack thereof) of these interventions. For young customers, pharmacy access seems important, yet is it only well-off young people and those living in urban areas who take advantage of these services?

Our own research in peri-urban Coastal Kenya found young people reported contraception purchased at a pharmacy to be ‘cheaper’ than going to a health facility. Transport expenses, waiting time, and payment for provider-ordered tests made ‘free’ contraception services at public facilities surprisingly costly.^11^ However, even in settings where pharmacy purchases are objectively more expensive, we posit that, with the exception of the very poorest, young users with less access to money will elect to absorb this financial cost. For them, contraceptives purchased from private sources may still be less ‘costly’ than the financial and especially the *non-financial* costs of travelling to and being seen accessing services in a public health facility. Young users, therefore, may appear willing to shoulder the out-of-pocket cost of contraception in pharmacies, while still being disproportionately burdened (as compared with adults) by the expense.

Research to understand young people’s willingness to pay and their sources of income is needed, especially given that this population group is likely to be financially dependent on other household members. As contraceptive services are shared to private retail pharmacies, we urge caution to ensure that disproportionate financial burden does not shift to the consumer, even those groups who appear to be enthusiastic adopters.

## Reaching ‘quality’ youth-friendly health services in pharmacies

The enthusiasm for self-care as it relates to contraception services and the role that pharmacies can play is not unwarranted. In many countries, private pharmacies are already an integral part of the network of providers relied on by young people. Nonetheless, there is still much to be done to improve the current provision of contraception services to young people in pharmacies and drug shops around the world. As such, we agree with Narasimhan and colleagues’ assertion that ‘self-care’ (provided through pharmacies or otherwise) is not a ‘shortcut’ for countries to achieve universal health coverage.^12^

Making additional contraception services available in pharmacies cannot come without improved regulation and control of pharmacy services as well as multisectoral efforts to improve demand for and delivery of quality services. Providing contraception through pharmacies can overcome important barriers to accessibility and acceptability for young people. It does not, however, absolve the health system and governments of their responsibility to ensure young people are receiving appropriate and effective and affordable contraceptive services.
